# Comparison of the Analgesic Effect of Paracetamol and Magnesium Sulfate during Surgeries

**Published:** 2016-09

**Authors:** Navid Kalani, Mohammad Sadegh Sanie, Hasan Zabetian, Mohammad Radmehr, Reza Sahraei, Hossein Kargar Jahromi, Hadi Zare Marzouni

**Affiliations:** 1 Medical Ethic Research Center, Jahrom University of Medical Sciences, Jahrom, Iran;; 2 Department of Anaesthesiology, Jahrom University of Medical Sciences, Jahrom, Iran;; 3 Research Center For Social Determinants Of Health, Jahrom University of Medical Sciences, Jahrom, Iran;; 4 Student Research Committee, Mashhad University of Medical Sciences, Mashhad, Iran

**Keywords:** Paracetamol, Magnesium sulphate, Analgesia, Pain, Limb

## Abstract

**BACKGROUND:**

New drugs are increasingly used to induce analgesia during surgeries. This study compared the analgesic effects of paracetamol and magnesium sulfate.

**METHODS:**

Sixty patients with American Society of Anesthesiologists (ASA) class I or II patients who were candidates for surgery of the lower limbs were randomly divided into three equal groups who were age and gender matched. Group 1 received paracetamol, and group 2, the magnesium sulfate during surgery and group 3 as the control. Pain intensities were measured and recorded using the Visual Analog Scale before surgery, in the recovery room, and 6, 12, and 18 hours after surgery.

**RESULTS:**

Pain intensities (7.10, 5.80, and 4.10) were higher in the control group; 6, 12, and 18 hours after surgery compared to the paracetamol (6.45, 4.15, 2.50) and the magnesium groups (7.25, 4.55, and 2.05), but the difference was not statistically significant.

**CONCLUSION:**

Paracetamol and magnesium sulfate were shown to have postoperative analgesic effects and reduce the quantity of narcotic use after surgery.

## INTRODUCTION

Pain is considered as the fifth vital sign that should be controled to prevent postoperative complications.^1^ Postoperative pain can lead to fundamental changes in body metabolism in susceptible patients resulting into alteration in blood pressure, causing cardiac ischemia, and respiratory, digestive and renal problems, may raise mortality rates, and increase hospital stay and treatment costs for operated patients. Factors such as race, age, gender, expressing the pain, underlying diseases, level of knowledge of physicians and their fear of causing complications, usually can affect the pain in patients.^2^

Several studies were conducted on pain reduction in operated patients, and attempts were made to quantify pain using Numeric Rating Scale (NRS) and Visual Analog Scale (VAS).^3^ One of medications that have attracted interest in pain control is intravenous use of acetaminophen (paracetamol).^4^ As intravenous injection of Non-Steroid Anti-inflammatory drugs (NSADIs) are unavailable in Iran (Removal of diclofenac from Iranian Pharmacopeia), and based on complications reported by tramadol (nausea, vomiting, convulsion, etc.), paracetamol may be a suitable substitute. Paracetamol is an acetaminophen product that is soluble in water and can be injected intravenously when there is a need for a strong effect and rapid onset of analgesic action. Propacetamol is a form of paracetamol that is hydrolyzed to paracetamol in blood, with analgesic and antipyretic effects, without substantial complications at the recommended doses (1-2 g).^5^

The mechanism of action of paracetamol is to suppress the synthesis of prostaglandins. Cyclooxygenase (COX) is the first enzyme in the production cycle of prostaglandins, and paracetamol blocks this cycle and acts as an analgesic.^6^ Nowadays, use of opioids is one of the main pillars of pain treatment.^7^ However, because of its side effects, there is a need to find more effective products for relieving pain and reducing use of opioids. NSAIDs and acetaminophen, which are usually used as antipyretic drugs, can be utilized as substitutes for, or a complement to, narcotic drugs to control postoperative pain.^8^

Considering the use of analgesia and the complications that may happen such as weakened respiration, physical dependence, and addiction, attempts were undertaken to introduce new drugs as analgesic substitutes, such as magnesium, which is a serum electrolytes and an N-methyl-diaspartate receptor antagonist^9^ with many clinical applications in preeclampsia, eclampsia, bronchospasm, and control of muscle spasm in tetanus; while it is used as an antidysrhythmic drug too.^10^

Moreover, magnesium is known as a natural blocker of calcium channels with analgesic effects. Furthermore, some studies have indicated that magnesium can enhance the effects of morphine in patients with chronic pain, that it is an N-methyl-diaspartate receptor antagonist for ion channels, preventing central and peripheral sensitization resulted from environmental stimuli. It can terminate increased sensitivity that was previously appeared.^9^ Substances that block calcium channels and are N-methyl-diaspartate receptor antagonist can be effective in preventing the emergence of pain and in its control. Little information is available on the role of magnesium in elimination of pain in man, and the study that has been conducted on the analgesic effects of magnesium in man has controversial result.^11^

The effect of magnesium were first recognized in treating arrhythmia and toxemia of pregnancy, and recently researchers have noticed its anesthetic and analgesic effects.^12^ Moreover, magnesium sulfate is used as a pharmacological agent in a range of clinical situations such as tachyarrhythmia, myocardial ischemia, asthma, preeclampsia, tocolysis and chills after surgery.^13^ The first mechanism explaining the analgesic effect of magnesium sulfate is its antagonistic effect on N-methy-diaspartate receptor. Stimulation of this receptor can increase the membrane permeability for potassium and calcium ions. Magnesium sulfate was shown to suppress the passage of electrical currents through membranes by suppressing the mentioned receptors.^14^

Magnesium sulfate has specific effects on vascular dilatation that is mediated through releasing vascular endothelium derived nitric oxide (NO=nitric oxide).^15^ The value and importance of magnesium sulfate as an adjuvant medication in anesthesia and as a inexpensive drug in postoperative analgesia has previously been confirmed.^14^ Considering complications caused by narcotics including bradycardia, respiratory depression, and fall in blood pressure, and as long-term use of narcotics is not always possible due to a dependence caused by it, anesthesia and pain specialists have searched for new products to reduce the use of narcotics for relieving pain in surgery that may be a combination of non-opioid pain- relieving compounds and narcotics.^16^

Several studies were conducted in this relation^14^^-^^16^ on various substances together with narcotics, but the required doses, the extent of pain reduction, and the possibility of using these substances in surgery have not been explained. Therefore, there is a need for research on the extent of analgesia caused by drugs of interest such as paracetamol (intravenous acetaminophen), and intravenous magnesium sulfate. This study compared the effect of intravenous paracetamol and magnesium sulfate before surgery on the extent of pain du. Ring and after surgery.

## MATERIALS AND METHODS

The convenient sampling method was used to select subjects from all patients referred to the Peymanieh Hospital in Jahrom, Southern Iran. All subjects were candidates for surgery in lower limbs and received spinal anesthesia and their blood pressure, respiratory and cardiac rates were recorded. There were three study groups. Group one received 15 mg of marcaine (3 ml of 0.5% marcaine) together with intravenous paracetamol (15 mg/kg body weight in 100 ml of 0.9% sodium chloride). Group two received 3 ml of 0.5% marcaine (15 mg/kg) together with 8 mg intravenous magnesium sulfate/kg body weight/hour until the completion of the surgery as an infusion dose. Group three, the control group, received 3 ml of 5% marcaine (15 mg/kg) together with 100 ml of normal saline as a placebo. It was estimated marcaine to be remained effective for about 2 hours. Twelve-lead electrocardiography was performed for all patients. The anesthesiologist used a 25 gauge spinal needle to inject the prepared solution in a sitting position. If the sensory block was inadequate, general anesthesia was induced, and the patient was excluded from the study. 

During the surgery, hemodynamic changes were measured every 5 minutes. Moreover, complications of spinal anesthesia including nausea, vomiting, fall of systolic blood pressure by more than 20% from the baseline, respiratory depression during surgery, and reduction in heart rate/minute by more than 20%, were recorded. If during surgery, drugs or measures other than routine care were required to eliminate complications and relieve pain, the patient was excluded from the study too. The inclusion criteria were (i) American Society of Anesthesiologists (ASA) class I or II, (ii) Not banned from undergoing spinal anesthesia, (iii) Not addicted to narcotics, (iv) Not taking psychiatric medications, (v) Not experiencing delirium, (vi) No history of hypertension, (vii) No history of heart problems, (viii) No drug allergies and (ix) older than 18 and less than 75 years of age. 

The exclusion criteria were (i) Allergies to drugs used in the study, (ii) Any unpredicted event during surgery endangering the patient’s life and hemodynamic status, (iii) Spinal anesthesia being changed to general anesthesia, (iv) History of alcohol abuse, recent use of tranquilizers, psychotropic and antipsychotic drugs, calcium channel blockers, and history of neuromuscular diseases, (v) Thyroidal disorders, history of any type of renal failure, underlying bradycardia (fewer than 60 heart beats per minute) and (vi) QRS interval prolongation (>0.12 second), QTc interval prolongation (>0.44 second).

At the completion of surgery, when the patient was conscious, hemodynamic signs (including blood pressure, and number of heart beats and breaths per minute) were measured, and they were evaluated during 24 hours period after surgery for emergence of sensory motor complications. 

The nurses and the doctors provided identical care, which was customary in the ward, for all three groups, and all the analgesics that were routinely used in the ward were given to the patients of all three groups. Pain intensity was recorded four times, first in the recovery room and then 6, 12, and 18 hours after surgery to obtain average pain intensity. If any of the patients in the ward felt severe pain, pethidine was administered and the quantity was recorded in a questionnaire sheet. The placebo group did not receive any medications and if they felt pain, pethidine was prescribed and its amount was recorded in the questionnaire sheet. Information was analyzed using descriptive statistical indices such as means and percentages, and statistical tests including repeated measurement and ANOVA were performed using SPSS software (Version 21, Chicago, IL, USA). 

## RESULTS

The average age of the subjects in the three groups was 36.6 years, while the majority were 20 years old. There were 23 female and 37 male participants: Seven females and 13 males in the control group, 8 females and 12 males in the paracetamol group, and 8 females and 12 males in the magnesium sulfate group. Thirty-two patients in the three groups underwent tibial surgeries and 28 for femoral operations. In the control group, 10 patients had tibial and eight femoral surgeries. In the paracetamol group, 10 members had tibial and 10 femoral operations. Finally, 10 subjects in magnesium sulfate group had tibial and 10 femoral surgeries.

The pain intensity before surgery determined by VASD table showed that the average pain intensity before surgery was 6.60, 6.70, and 7.55 in the control, paracetamol, and magnesium sulfate groups, respectively. There were no significant differences between the three groups for pain intensity before surgery (*p*>0.05). In the recovery room, the average pain intensy determined by VASD table was 1.20, 1.35, and 1.00 in the control, paracetamol, and magnesium sulfate groups, respectively. There were no significant differences between the three groups regarding pain intensity in the recovery room (*p*>0.05). Based on the VASD table, the average pain intensity was 7.10, 6.45, and 7.25 in the control, paracetamol, and magnesium sulfate groups, respectively, 6 hours after surgery. There were no significant differences between the 3 groups for average pain intensity, 6 hours after surgery (*p*>0.05). 

The mean of pain intensity 12 hours after surgery, as determined by VASD table, was 5.80, 4.15, and 4.55 in the control, paracetamol, and magnesium sulfate groups, respectively. There were no significant differences between the 3 groups for average pain intensity, 12 hours after surgery (*p*>0.05). Finally, based on the VASD table, the average pain intensity in the control, paracetamol, and magnesium sulfate groups were 4.10, 2.50, and 2.05, respectively. there were no significant differences between the 3 groups with respect to the average pain intensity, 18 hours after surgery (*p*>0.05). The comparison of pain intensity before surgery, in the recovery room, and 6, 12, and 18 hours after surgery in the three groups was shown in Table 1.

**Table 1:** Determination and comparison of average pain intensities before surgery, in the recovery room, and 6, 12, and 18 hours after surgery in the three groups

**Pain intensity**				
**Group**	**In recovery room**	**6 h after surgery**	**12 h after surgery**	**18 h after surgery**
Control	6.60	1.2	7.10	4.10
Paracetamol	6.70	1.35	6.45	2.50
Magnesium sulfate	7.55	1.00	7.25	2.05

The mean percentages of administerd narcotics in the control, paracetamol, and magnesium sulfate groups were 33.3% (30% pethidine and 3.3% morphine), 25% (pethidine), and 13.3% (pethidine), respectively. The difference between the three groups was not statistically significant. The comparison of respiratory changes in the 3 groups in the recovery room showed that the mean respiratory rate/minute was 18.05, 18.66, and 18.70 in the control, paracetamol, and magnesium sulfate groups, respectively. There were no significant differences between the three groups for respiratory rate/minute in the recovery room (*p*>0.05).

The average systolic blood pressure values determined for the 3 groups in the recovery room were 121.85, 116.33, and 115.39 in the control, paracetamol, and magnesium sulfate groups, respectively. Comparison of the percentages of narcotics used in the three groups after surgery was shown in table 2.

**Table 2:** Comparison of the percentages of narcotics used in the three groups after surgery

**Group**	**After surgery**			
	**0**	**Fenta**	**Morphine**	**Pethedine**
	**%**	**%**	**%**	**%**
Control	0.0	0.0	3.3	30.0
Paracetamol	8.3	0.0	0.0	25.0
Magnesium sulfate	20.0	0.0	0.0	13.3

The groups were not significantly different for average systolic blood pressure in the recovery room (*p*>0.05). The average number of heart rate/minute in the three groups in the recovery room was 81.20, 84.25, and 81.65, in the control, paracetamol, and magnesium sulfate groups, respectively. There were no significant differences between the three groups for average number of heart rate/minute (*p*>0.05). Nausea, vomiting, and any signs of itching were also determined in the recovery room. Nausea was observed in six patients in the control, in 2 patients in the paracetamol, and in 5 patients in the magnesium sulfate groups. The groups were not significantly different with respect to nausea in the recovery room (*p*>0.05). No instances of vomiting or itching were observed in any of the three groups. 

## DISCUSSION

Our study showed that use of intravenous paracetamol (15 mg/kg body weight in 100 mm of 0.9% sodium chloride) could have analgesic effects in patients and reduce the required quantity of narcotics after surgery. Moreover, this research indicated that the use of magnesium sulfate (8 mg/kg body weight once every hour during the surgery) could have analgesic effects in patients and reduce the amount of narcotics after surgery, but the differences were not statistically significant. 

The scores on pain intensity in patients using the VAS system, and based on narcotic use after surgery, showed that paracetamol and magnesium sulfate could reduce the pain intensity, 6, 12, and 18 hours after operation, but there were no significant differences between the the 3 groups. Nevertheless, pain intensity in the paracetamol group, 12 after the surgery was lower than magnesium sulfate group. The pain intensity was lower in the magnesium sulfate group, 18 hours after the surgery when compared to the paracetamol group. Therefore, magnesium sulfate had longer analgesic effects in comparison to paracetamol. The two groups were not significantly different with respect to pain reduction. For comparison of the paracetamol and magnesium sulfate groups in relation to the quantities of narcotics used after surgery, the two groups were not significantly different, but the quantity of narcotics after surgery in the magnesium sulfate group was less than the paracetamol group.

Our findings revealed that magnesium sulfate resulted into a reduction in narcotics use after surgery when compared to paracetamol even the difference was not statistically significant. Scoring of pain by two groups receiving paracetamol and intravenous morphine after orthopedic surgery was previously undertaken. Comparison of the difference between two groups revealed no significant difference too similar to our study. The quantity of narcotic used in the paracetamol group was less in comparison to the control group, showing a decrease for narcotics in these patients.^17^

In research carried out on the effects of paracetamol on pain intensity after appendectomy operation in comparison to pethidine, it was found that pain intensity in the paracetamol group was significantly less,^18^ and these results were identical to our research. In another study conducted on the effects of acetaminophen and paracetamol on pain intensity in patients after orthopedic surgery, it was noted that scores given to pain intensity felt by patients decreased significantly, and the need for narcotics also declined significantly in comparison to the control group. These findings were the same as our study even their results denoted to a significant difference between groups.^19^

A study was carried out in Russia in 2002 on analgesic effect of paracetamol after surgery on 30 patients demonstrating that paracetamol reduced the pain intensity after surgery.^20^ In another study that was conducted by Amiundertaken on 60 patients undergoing spinal injury to compare the analgesic effects of tramadol together with paracetamol and tramadol, it was observed that the analgesic effect was greater in the paracetamol group.^21^ In all of the mentioned studies similar to ours, the number of injections and the total dose of narcotics used declined significantly. 

The effects of intravenous magnesium sulfate on pain intensity after the surgery in 68 patients undergoing elective cesarean section under spinal anesthesia, magnesium sulfate could reduce the pain intensity even the difference was not statistically significant. However, the quantity of narcotics needed after surgery in the control group was greater compared to the magnesium sulfate group which is similar to our findings.^22^ In another research carried out on the postoperative effects of magnesium sulfate injection during inguinal hernia operation, it was found that pain intensity significantly decreased after surgery and the quantity of narcotics used after the operation in the magnesium sulfate group was less than the control group which is identical to our results.^23^

In a study on the effect of magnesium sulfate injection for postoperative pain in patients undergoing thoracotomy, it was reported that the scores given to pain intensity declined in the magnesium sulfate group and the quantity of needed narcotics in this group was less than the control group confirming our study.^24^ The effect of magnesium sulfate on the quantity of needed narcotics in 81 patients after surgery was investigated and was shown that magnesium sulfate reduced pain intensity and the amount of required narcotics after surgery while these findings are the same as our results.^25^ A research conducted on 40 patients who were candidates for total hip joint replacement, the analgesic effect of magnesium sulfate was assessed and was demonstrated that the pain intensity and quantity of required narcotics were less in the magnesium sulfate group when compared to the controls similar to our study.^26^

In our research, complications such as suppressed respiration, vomiting, and itching were not reported in any of the patients, but nausea was observed in some patients of all three groups. However, no significant correlation was found. In previous studies, in most cases, side effects in the paracetamol and magnesium sulfate groups were not different from those in other groups but, in general, these side effects were fewer in the paracetamol and magnesium sulfate groups and no serious complications were seen in any of the groups.^22^^-^^26^ Reduced complications in the paracetamol and magnesium sulfate groups can be attributed to the less quantity of narcotics needed by these groups after surgery. Lack of complications in our study can be attributed to the low dose of injected narcotics and to the prescription of the anti-nausea drug metoclopramide at the start of surgery.

Our study indicated that intravenous paracetamol had postoperative analgesic effects and could reduce the number of injections and the total dose of injected narcotics. Therefore, intravenous paracetamol can be used as a suitable adjuvant anesthetic for narcotics. Moreover, intravenous magnesium sulfate had postoperative analgesic effect and reduced the number of injections and the total dose of injected narcotics too. Furthermore, magnesium sulfate had longer analgesic effects compared to paracetamol and caused greater decrease in the quantity of required narcotics compared to paracetamol, even the difference was not statistically significant. Another important point was that the average ages in the three groups were not significantly different, which increased the value of comparison of the variables among the three groups. In addition, the three groups were similar regarding gender.

Paracetamol and magnesium sulfate were shown to have postoperative analgesic effects and reduce the quantity of required narcotics after surgery. So these drugs could be recommended to decrease the amount of required narcotics and can be used as adjuvant drugs to reduce pain. 

## References

[1] Ribeiro NC, Barreto SC, Hora EC, de Sousa RM (2011). The nurse providing care to trauma victims in pain: the fifth vital sign. Rev Esc Enferm USP.

[2] Weibel S, Jokinen J, Pace NL, Schnabel A, Hollmann MW, Hahnenkamp K, Eberhart LH, Poepping DM, Afshari A, Kranke P (2016). Efficacy and safety of intravenous lidocaine for postoperative analgesia and recovery after surgery: a systematic review with trial sequential analysis. Br J Anaesth.

[3] Hawker GA, Mian S, Kendzerska T, French M (2011). Measures of adult pain: Visual Analog Scale for Pain (VAS Pain), Numeric Rating Scale for Pain (NRS Pain), McGill Pain Questionnaire (MPQ), Short-Form McGill Pain Questionnaire (SF-MPQ), Chronic Pain Grade Scale (CPGS), Short Form-36 Bodily Pain Scale (SF-36 BPS), and Measure of Intermittent and Constant Osteoarthritis Pain (ICOAP). Arthritis Care Res (Hoboken).

[4] Jean R, Ducasse JL, Montastruc JL (2001). Treatment of acute renal colic in a French emergency department: A comparison of simulated cases and real cases in acute pain assessment and management. Eur J Clin Pharmacol.

[5] Tzortzopoulou A, McNicol ED, Cepeda MS, Francia MB, Farhat T, Schumann R (2011). Single dose intravenous propacetamol or intravenous paracetamol for postoperative pain. Cochrane Database Syst Rev.

[6] Dejonckheere M, Desjeux L, Deneu S, Ewalenko P (2001). Intravenous tramadol compared to propacetamol for postoperative analgesia following thyroidectomy. Acta Anaesthesiol Belg.

[7] Cura Della Redazione A (2016). WHO analgesic pain ladder and weak opioids. Assist Inferm Ric.

[8] da Costa BR, Reichenbach S, Keller N, Nartey L, Wandel S, Jüni P, Trelle S (2016 ). Effectiveness of non-steroidal anti-inflammatory drugs for the treatment of pain in knee and hip osteoarthritis: a network meta-analysis. Lancet.

[9] Ghannoum M, Kazim S, Grunbaum AM, Villeneuve E, Gosselin S (2016). Massive acetaminophen overdose: effect of hemodialysis on acetaminophen and acetylcysteine kinetics. Clin Toxicol (Phila).

[10] Barman PK, Mukherjee R, Prusty BK, Suklabaidya S, Senapati S, Ravindran B (2016). Chitohexaose protects against acetaminophen-induced hepatotoxicity in mice. Cell Death Dis.

[11] van der Marel CD, Peters JW, Bouwmeester NJ, Jacqz-Aigrain E, van den Anker JN, Tibboel D (2007). Rectal acetaminophen does not reduce morphine consumption after major surgery in young infants. Br J Anaesth.

[12] Warner TG, Mitchell JA (2002). Cyclooxygenase-3 filling in the gaps toward a cox continuum. Proc Natl Acad Sci.

[13] Barden J, Edwards JE (2002). Single-dose paracetamol for acute post-operative pain in adults Aquanitive systemic review. BMC Anesthesiology.

[14] Baaklini LG, Arruda GV, Sakata RK (2015). Assessment of the Analgesic Effect of Magnesium and Morphine in Combination in Patients With Cancer Pain: A Comparative Randomized Double-Blind Study. Am J Hosp Palliat Care.

[15] Srebro DP, Vučković S, Vujović KS, Prostran M (2014). Anti-hyperalgesic effect of systemic magnesium sulfate in carrageenan-induced inflammatory pain in rats: influence of the nitric oxide pathway. Magnes Res.

[16] White PF (2005). The changing role of non-opioid analgesic techniques in the management of postoperative pain. Anesth Analg.

[17] Delbos A, Boccard E (1995). The morphine-sparing effect of propacetamol in orthopedic postoperative pain. J Pain Symptom Manage.

[18] Kolahdouzan K, Eydi M, Mohammadipour Anvari H, Golzari SE, Abri R, Ghojazadeh M, Ojaghihaghighi SH (2014). Comparing the efficacy of intravenous acetaminophen and intravenous meperidine in pain relief after outpatient urological surgery. Anesth Pain Med.

[19] Sinatra RS, Jahr JS, Reynolds LW, Viscusi ER, Groudine SB, Payen-Champenois C (2005). Efficacy and safety of single and repeated administration of 1 gram intravenous acetaminophen injection (paracetamol) for pain management after major orthopedic surgery. Anesthesiology.

[20] Nikoda VV, Maiachkin RB (2002). Study of analgesic efficacy of propacetamol in the postoperative period using a double blind placebo controlled method. Anesteziol Reanimatol.

[21] Emir E, Serin S, Erbay RH, Sungurtekin H, Tomatir E (2010). Tramadol versus low dose tramadol-paracetamol for patient controlled analgesia during spinal vertebral surgery. Kaohsiung J Med Sci.

[22] Davoudi M, Tahmasebi R, Zolhavareih SM (2013). Evaluation of the effect of intravenous magnesium sulfate on postoperative pain after cesarean section under spinal anesthesia. Sci J Hamadan Univ Med Sci.

[23] Gousheh M, Pipelzadeh M, Akhondzadeh M, Olapour A, Alizadeh Z, Sahafi S (2013). Intraoperative Administration of Magnesium Sulfate on Post-Operative Pain of Inguinal Herniorrhaphy under General Anesthesia. Armaghane Danesh.

[24] Ozcan PE, Tugrul S, Senturk NM, Uludag E, Cakar N, Telci L, Esen F (2007). Role of magnesium sulfate in postoperative pain management for patients undergoing thoracotomy. J Cardiothorac Vasc Anesth.

[25] Telci L, Esen F, Akcora D, Erden T, Canbolat AT, Akpir (2002). Evaluation of effects of magnesium sulphate in reducing intraoperative anaesthetic requirements. Br J Anaesth.

[26] Hwang JY, Na HS, Jeon YT, Ro YJ, Kim CS, Do SH IV (2010). infusion of magnesium sulphate during spinal anaesthesia improves postoperative analgesia. Br J Anaesth.

